# Molecular and physiological mechanisms of drought tolerance in grapevine

**DOI:** 10.3389/fpls.2026.1944974

**Published:** 2026-08-25

**Authors:** Yongqiang Chen, Liqin Tu, Zhi Luo

**Affiliations:** 1 College of Horticulture and Forestry, Tarim University, Alar, China; 2 National and Local Joint Engineering Laboratory of Efficient and High-Quality Cultivation and Deep Processing Technology of Characteristic Fruit Trees in Southern Xinjiang, Alar, China; 3 Characteristic Forest and Fruit Technology Innovation Center of Southern Xinjiang in XPCC, Alar, China; 4 Xinjiang Production & Construction Corps Key Laboratory of Protected Agriculture, Alar, China

**Keywords:** abscisic acid, antioxidant defense, hydraulic segmentation, stomatal regulation, *Vitis vinifera* L., water deficit

## Abstract

Drought stress severely limits grapevine productivity and compromises fruit quality, with its adverse effects expected to intensify under ongoing climate change. This review synthesizes recent advances in elucidating the molecular and physiological mechanisms underlying drought tolerance in *Vitis vinifera*. Key physiological responses include abscisic acid (ABA)-mediated amplification of stomatal closure, osmotic adjustment, activation of antioxidant defense systems, and hydraulic vulnerability segmentation-a protective strategy involving the targeted sacrifice of petioles and basal leaves to maintain hydraulic integrity in stems and roots. These coordinated responses are regulated by a complex transcriptional network centered on NAC, WRKY, MYB, and bZIP transcription factors, and further modulated by hormone metabolism, potassium channel activity, aquaporin-facilitated water transport, and both enzymatic and non-enzymatic antioxidant pathways. We also identify critical knowledge gaps requiring urgent attention: (First) field-scale validation of transgenic or CRISPR-edited grapevine lines; (Second) the molecular basis of rootstock–scion signaling during drought stress; and (Third) the integration of multi-omics data with high-resolution phenotyping to accelerate precision breeding. Collectively, this review provides an integrative conceptual framework to support sustainable viticulture in water-limited environments.

## 1 Introduction

Drought stress represents a primary environmental constraint limiting grapevine productivity, geographical distribution, and fruit quality worldwide. With accelerating global climate change, the frequency, intensity, and duration of drought events are projected to increase-particularly in semi-arid and Mediterranean-type viticultural regions, thereby posing a significant threat to the long-term sustainability of grape production (Gupta et al., 2020; Zhang et al., 2022a; Kim et al., 2024; Sato et al., 2024).

Over the past two decades, drought tolerance mechanisms have been extensively characterized in model plant species, revealing a highly coordinated signaling network that integrates hormonal cues, hydraulic dynamics, and metabolic reprogramming (Gupta et al., 2020; Kim et al., 2024; Sato et al., 2024; Wang and Xu, 2026). The abscisic acid (ABA)-dependent and ABA-independent signal transduction pathways are among the best-characterized stress-response mechanisms in plants. Core components, including pyrabactin resistance (PYR/PYL) receptors, type 2C protein phosphatases (PP2Cs), and sucrose non-fermenting 1-related protein kinase 2 (SnRK2) kinases, have been comprehensively elucidated (Li et al., 2023). Furthermore, key transcription factor families-particularly dehydration-responsive element-binding protein/C-repeat-binding factor (DREB/CBF), NAC, and basic leucine zipper (bZIP) proteins-have been shown to coordinately regulate the expression of downstream stress-responsive genes (Yang et al., 2025; Chen and Xia, 2025). Nevertheless, grapevine (*Vitis vinifera* L.) poses distinct challenges for molecular investigation as a perennial woody species with an extended juvenile phase and a highly heterozygous genome. Consequently, it remains to be experimentally validated to which extent these evolutionarily conserved regulatory modules operate in grapevine and how they are modulated.

In recent years, significant advances have been made in deciphering the molecular basis of drought tolerance in grapevine, largely driven by breakthroughs in high-throughput sequencing technologies and molecular biotechnological tools. The release of multiple high-quality, chromosome-level reference genomes for grapevine (Shi et al., 2023; Wang et al., 2024; Guo et al., 2025), coupled with comprehensive multi-omics analyses, including transcriptomic, proteomic, and metabolomic profiling under controlled water-deficit conditions (Haider et al., 2017; Savoi et al., 2017; Ma et al., 2023), has greatly facilitated the identification and functional characterization of drought-associated genes. These efforts have uncovered a growing repertoire of pivotal genes spanning early signal perception, transcriptional reprogramming, and execution of physiological and biochemical adaptations. This review synthesizes recent progress in understanding grapevine drought tolerance, from initial stress sensing and signal transduction to downstream physiological adjustments, thus providing an integrated framework of the underlying regulatory networks.

## 2 Physiological responses to drought stress

**Figure 1:**
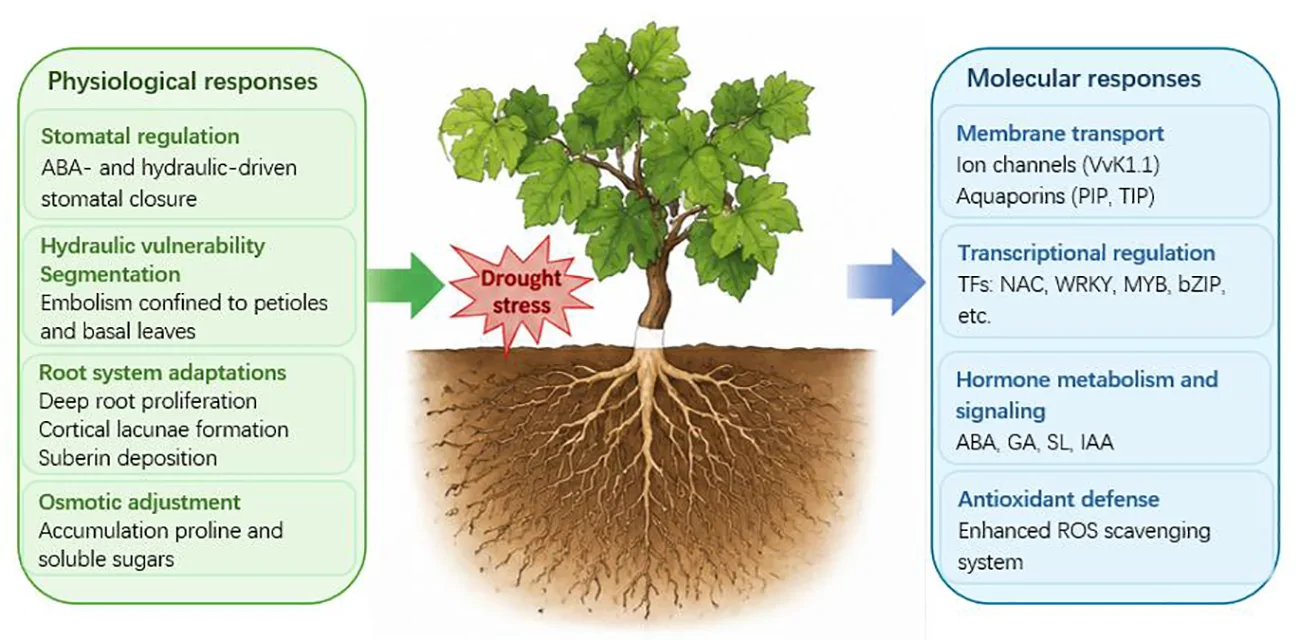
Overview of physiological and molecular responses to drought stress in grapevine. Physiological adaptations encompass: ABA- and hydraulic signal-mediated stomatal closure to limit transpirational water loss; hydraulic vulnerability segmentation, which confines xylem embolism to expendable organs such as petioles and basal leaves; root architectural and functional modifications, including deep root proliferation, cortical lacunae formation, and suberin deposition; and osmotic adjustment via accumulation of compatible solutes, notably proline and soluble sugars. At the molecular level, drought responses involve: modulation of membrane transport through ion channels (e.g., VvK1.1) and aquaporins (PIP and TIP families); transcriptional reprogramming orchestrated by key transcription factors such as *NAC*, *WRKY*, *MYB*, and *bZIP* families; dynamic regulation of phytohormone metabolism and signaling pathways [ABA, gibberellins (GA), strigolactones (SL), and auxin (IAA)]; and upregulation of the antioxidant defense system to scavenge reactive oxygen species (ROS). Together, these coordinated physiological and molecular mechanisms underpin grapevine tolerance to water deficit.

In grapevine, stomatal closure represents the most immediate physiological response to drought stress. This response is initially triggered by hydraulic signals and subsequently sustained by ABA (**Figure 1**), serving to minimize transpirational water loss while maintaining an optimal balance with carbon assimilation (Tombesi et al., 2015; Rodriguez-Dominguez et al., 2016; Gambetta et al., 2020; Dayer et al., 2021). Beyond rapid short-term adjustments, stomatal behavior is also subject to seasonally coordinated regulation with marked plasticity: stomatal sensitivity declines progressively over the growing season, enabling leaves to function at increasingly lower water potentials (Herrera et al., 2022). Under drought, plants accumulate osmoprotectants such as proline and soluble sugars (Ozturk et al., 2021), which maintain cellular turgor and stabilize macromolecules against dehydration damage. Concurrently, enhanced antioxidant enzyme activities and elevated non-enzymatic antioxidants, such as ascorbate and glutathione (**Figure 1**), synergistically scavenge drought-induced reactive oxygen species (ROS) to alleviate oxidative damage. Transgenic studies consistently corroborate these biochemical adaptations: drought-tolerant lines display higher proline accumulation, greater antioxidant enzyme activity, and significantly lower levels of MDA and hydrogen peroxide (H_2_O_2_) compared with drought-sensitive controls (Ju et al., 2020a; Chen et al., 2021; Cui et al., 2023).

Additionally, hydraulic vulnerability segmentation serves as a key protective strategy for perennial stems by localizing xylem embolism to expendable organs, such as petioles and basal leaves (**Figure 1**). Notably, basal leaves exhibit greater hydraulic vulnerability than apical leaves, facilitating the preferential abscission of older, less photosynthetically efficient foliage under severe drought stress (Brodersen et al., 2013; Hochberg et al., 2017). Once embolized, petioles fail to restore hydraulic conductivity even following rehydration, confirming that leaf shedding constitutes an irreversible, terminal acclimation response (Gambetta et al., 2020). Simultaneously, root system adaptations, including deep root proliferation, cortical lacunae formation, and enhanced suberin deposition (**Figure 1**), improve water uptake efficiency while minimizing uncontrolled water loss to drying soil (Alsina et al., 2011; Hochberg et al., 2016; Cuneo et al., 2021). Collectively, these integrated physiological strategies enhance grapevine resilience during water scarcity and facilitate rapid physiological recovery upon rewatering.

## 3 Sensing and early signaling of drought stress in grapevine

**Figure 2:**
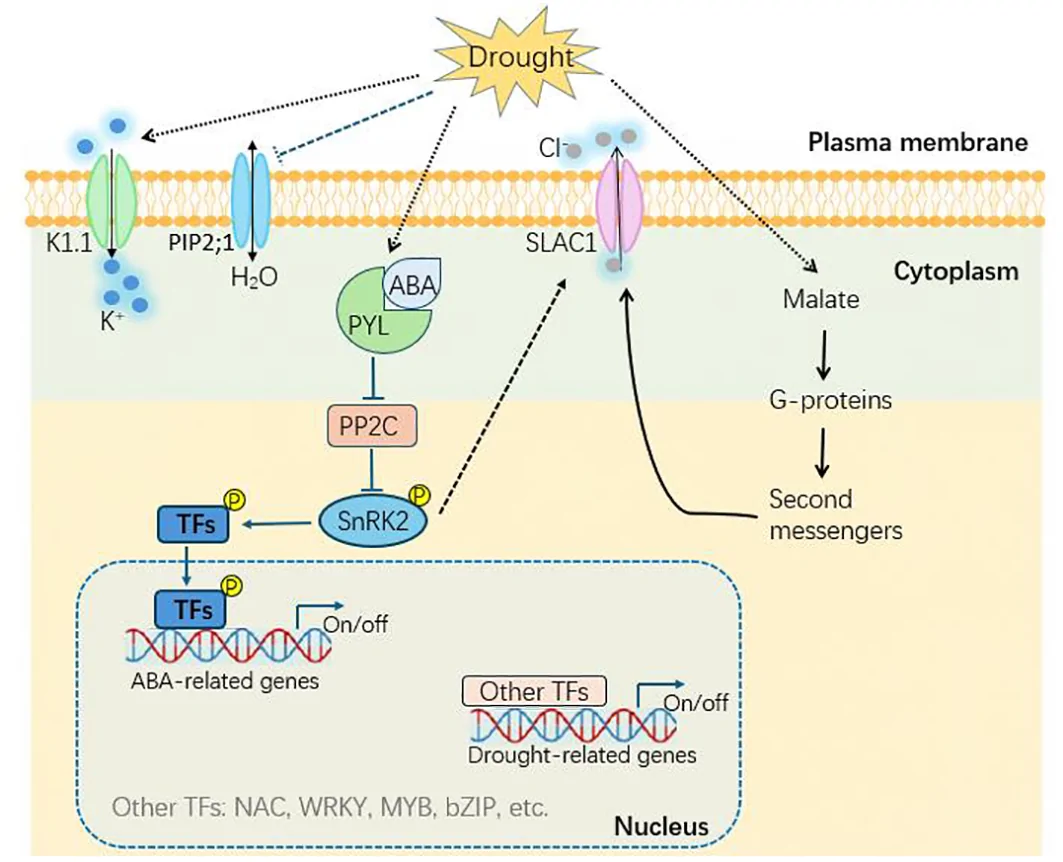
Drought perception and transcriptional regulation of drought-responsive genes in grapevine. Drought stress induces ABA accumulation, which is perceived by PYL receptors. This interaction inhibits PP2C phosphatases, leading to the activation of SnRK2 kinases. Activated SnRK2s phosphorylate AREB/ABF transcription factors, which subsequently orchestrate the expression of ABA- and drought-responsive genes. Other transcription factors, including members of the NAC, WRKY, MYB, and bZIP families, are also integral to transcriptional reprogramming during drought stress. Concurrently, the drought-responsive metabolite malate is sensed by heterotrimeric G-proteins, which modulate the production of second messengers such as Ca²^+^, cAMP, and IP_3_. This signaling cascade triggers an elevation of cytosolic Ca²^+^ concentration, leading to the activation of the S-type anion channel SLAC1. SLAC1 activation promotes anion efflux, resulting in turgor loss that drives stomatal closure. In parallel, aquaporins, such as VvPIP2;1, facilitate transmembrane water transport to sustain cellular water homeostasis, while potassium channels mediate K^+^ influx and contribute to ion homeostasis; Both aquaporin and potassium channel activities are dynamically regulated to coordinate cellular responses.

The initial perception of water deficit in grapevine initiates a cascade of hydraulic and chemical signals that orchestrate downstream adaptive responses. A pivotal early event is the decline in leaf water potential, triggering passive, hydraulically driven stomatal closure, occurring prior to any substantial increase in ABA concentration (Hochberg et al., 2013; Tombesi et al., 2015; Degu et al., 2019; Marusig and Tombesi, 2020). Meanwhile, ABA serves as the central signaling molecule that reinforces and sustains stomatal closure over the long term, thereby preventing stomatal reopening upon rewatering (Tombesi et al., 2015). The ABA signaling pathway in grapevine has been systematically characterized: nine PYR/PYL ABA receptors, 85 PP2Cs, seven SnRK2s, and eight ABA-responsive element (ABRE)-binding factors (ABFs) have been identified (Pilati et al., 2017; Zhang et al., 2021). Among these, functional validation in grapevine has been achieved for certain members. For the PYR/PYL family, overexpression of *VaPYL4* from *V. amurensis* in *Arabidopsis* and grape calli enhances tolerance to multiple abiotic stresses (Ren et al., 2022). For the SnRK2 family, *VvSnRK2.7* overexpression in Arabidopsis increases antioxidant enzyme activities and reduces membrane damage under drought (Lan et al., 2024). For PP2Cs, several members (e.g., VvPP2C4, VvPP2C9, VvPP2C59, VvPP2C60, and VvPP2C66) have been identified as ABA receptor interactors or drought-responsive regulators (Boneh et al., 2012; Zhang et al., 2021). For ABF transcription factors, VvABF2 and VvABF8 have been functionally implicated in osmotic and drought stress responses (Liu et al., 2019; Zhang et al., 2021). Although the core ABA signaling modules are evolutionarily conserved across plant species (**Figure 2**), their specific functional contributions and detailed mechanistic insights to drought tolerance in grapevine remain largely uncharacterized.

Beyond ABA, early drought-induced signals, including ROS bursts, calcium (Ca²^+^) signatures, and specific tricarboxylic acid (TCA) cycle metabolites such as malate, contribute to drought perception and signaling (Dubrovina et al., 2015; Mimata et al., 2026). Notably, malate promotes stomatal closure via a G-protein–dependent pathway that triggers cytosolic Ca²^+^ elevation and subsequently activates the guard-cell anion channel Slow Anion Channel-associated 1 (SLAC1) (Mimata et al., 2026) (**Figure 2**). In *Arabidopsis* and other model plants, SLAC1 is phosphorylated and activated by SnRK2 kinases, suggesting that this regulatory mechanism may be conserved in grapevine (Geiger et al., 2009; Takahashi et al., 2020). Together, these early hydraulic and chemical signals constitute a rapid sensory network that relays water-deficit signals to cellular effectors, thereby initiating extensive transcriptional and metabolic reprogramming.

Regulation of membrane transport processes is essential for maintaining cellular ion homeostasis and water balance during drought stress. The grapevine Shaker-type inward-rectifying K^+^channel, VvK1.1, an ortholog of Arabidopsis AKT1 (**Figure 2**), is strongly induced in grape berries under drought stress and localizes specifically to the phloem vasculature and seed coat (pip teguments). Its activity is regulated by the calcineurin B-like calcium sensor CBL1–protein kinase CIPK23 signaling module. Notably, VvK1.1 activates at more negative membrane potentials, and is highly responsive to both drought stress and ABA (Cuéllar et al., 2010). Grapevine aquaporins (AQPs) also serve as pivotal regulators of plant water status (**Figure 2**). The expression patterns of these AQPs (e.g., VvPIP2;1 and VvTIP2;1) were significantly correlated with stomatal conductance, suggesting their functional involvement in modulating leaf gas exchange under water-deficit conditions (Braidotti et al., 2024). These early signals converge to activate transcriptional and metabolic programs, as well as regulate membrane transport proteins, thereby maintaining cellular ion homeostasis and water balance. For instance, ABA signaling reinforces hydraulic-driven stomatal closure via the SnRK2–AREB/ABF module, upregulating osmoprotectant and antioxidant genes.

## 4 Drought-responsive regulatory networks in grapevine

### 4.1 Predominant transcription factors

Transcription factors (TFs) serve as central orchestrators of drought-induced physiological and molecular responses in grapevine. Functional characterization has identified members of several major TF families, including NAC, WRKY, MYB, and bZIP, as key regulators of drought tolerance. Transcriptomic studies have revealed that thousands of genes exhibit differential expression in grapevine tissues under drought stress, with a substantial proportion encoding TFs (Haider et al., 2017). Among these, NAC, WRKY, MYB, and bZIP family members have been experimentally validated (**Figure 2**), acting either as positive or negative regulators of drought adaptation.

NAC TFs function predominantly as positive regulators of drought tolerance in grapevine. Overexpression of *VvNAC17* significantly enhances drought resilience, as demonstrated in transgenic Arabidopsis and grape callus lines. These transgenic lines display reduced MDA accumulation, elevated activities of antioxidant enzymes such as superoxide dismutase and peroxidase, increased monomeric anthocyanin content, and upregulated expression of drought-responsive genes, including *VvDREB1A*, *VvDREB2A*, and *VvRD29A*. Notably, *VvNAC17* directly binds to the promoters of *VvDREB1A* and *VvUFGT* and activates their transcription (Ju et al., 2020b; Jin et al., 2025). Similarly, overexpression of *VvNAC08* in Arabidopsis improved drought survival, accompanied by reduced MDA and H_2_O_2_ levels, enhanced antioxidant enzyme activities, increased proline accumulation, and upregulation of ABA- and stress-responsive genes (Ju et al., 2020a). The wild species *Vitis amurensis* genes *VaNAC26* and *VaNAC17* enhance drought tolerance in transgenic *Arabidopsis* via modulation of jasmonic acid (JA) biosynthesis and signaling pathways (Fang et al., 2016; Su et al., 2020). More recently, *VvNAC33* was identified as a key positive regulator of drought tolerance, activating the antioxidant defense system in *Arabidopsis* to enhance ROS scavenging and alleviate oxidative damage (Xu et al., 2025).

WRKY TFs exhibit both positive and negative-regulatory roles in grapevine drought responses. Overexpression of *VvWRKY70* conferred enhanced drought tolerance in both transgenic *Arabidopsis* and grapevine seedlings. *VvWRKY70* directly binds to and activates the promoters of drought-responsive marker genes, including *VvRD22*, *VvRD29A*, *VvDREB2A*, and *VvERD14* (Li et al., 2026). Similarly, *VaWRKY14* from *V. amurensis* improved drought tolerance in transgenic *Arabidopsis*, with transgenic lines exhibited higher leaf water content and increased activities of antioxidant enzymes (Zhang et al., 2018). In contrast, *VvWRKY13* functions as a negative regulator: transgenic *Arabidopsis* lines overexpressing *VvWRKY13* displayed heightened sensitivity to drought stress, reduced proline and soluble sugar accumulation, diminished antioxidant enzyme activities, and increased ROS accumulation (Hou et al., 2020). Another negative regulator, *VvWRKY18*, promotes stomatal development and accelerates leaf water loss by downregulating key negative regulators of stomatal development (Zhang et al., 2022b).

MYB family TFs contribute to drought tolerance via diverse molecular mechanisms. *VvMYB2*, isolated from the drought-tolerant rootstock ‘Beta’, enhances salinity and drought tolerance in Arabidopsis by increasing proline and chlorophyll contents and upregulating the activities of peroxidase (POD), superoxide dismutase (SOD), and catalase (CAT) (Ren et al., 2023). VvMYC4-a, a bHLH/MYB-related transcription factor, positively regulates drought tolerance by directly activating the flavonol biosynthesis genes Flavonoid 3′-hydroxylase (*VvF3′H*) and flavonol synthase (*VvFLS*), thereby promoting flavonol accumulation and enhancing ROS scavenging capacity (Tan et al., 2025). In stomatal regulation, *VvMYB60* contributes to drought tolerance by modulating ABA-induced stomatal closure (Galbiati et al., 2011). Among bZIP transcription factors, overexpression of *VlbZIP36* in Arabidopsis improves drought tolerance by optimizing plant water status, primarily through reduced water loss and attenuated cellular damage (Tu et al., 2016). VvbZIP45 directly binds to the promoter of the annexin gene *VvANN1* and activates its expression under drought stress, thereby maintaining ROS homeostasis via suppression of MDA, H_2_O_2_, and superoxide anion (O_2_^-^) levels (Niu et al., 2023).

### 4.2 Hormone metabolism and signaling

Beyond ABA, modulation of phytohormone homeostasis also contributes critically to drought tolerance in grapevine (**Figure 1**). The gibberellin (GA) catabolic gene *VvGA2ox8*-Like is markedly induced under drought stress and functions as a positive regulator of drought tolerance. Transient overexpression of *VvGA2ox8-Like* in grape leaves reduces H_2_O_2_ and MDA accumulation while increasing proline content, antioxidant enzyme activities, and transcript levels of drought-responsive genes (Yang et al., 2026). These results establish a functional nexus linking GA catabolism, antioxidant defense activation, and secondary metabolism during drought stress.

Strigolactones (SLs) likewise play a role in conferring drought tolerance in grapevine (Wang et al., 2021, 2025). Carotenoid Cleavage Dioxygenase 7 (*VvCCD7*), which encodes a key enzyme in strigolactone biosynthesis, enhances drought tolerance when overexpressed in transgenic Arabidopsis. Transgenic lines exhibited elevated POD activity and increased proline accumulation. Moreover, overexpression of *VvCCD7* also conferred enhanced tolerance to low-phosphorus stress, indicating its broader role in abiotic stress resistance (Wang et al., 2025). In contrast, Gretchen Hagen 3-9 (*VvGH3-9*), an indole-3-acetic acid (IAA) amido synthetase, functions as a negative regulator of drought tolerance. *VvGH3-9*-overexpressing lines displayed significantly higher levels of IAA and ABA, increased relative electrolyte leakage, and elevated MDA content; conversely, proline accumulation and the activities of POD, SOD, and CAT were markedly reduced (Lu et al., 2022). In model plants, ABA interacts synergistically with JA, antagonistically with GA and cytokinins, and modulates auxin signaling to reshape root architecture under drought (Singh and Roychoudhury, 2023; Liao et al., 2025). However, in grapevine, the molecular basis of these hormonal interactions remains largely unexplored. Although individual hormone responses have been documented, how they converge with ABA signaling to coordinate drought tolerance awaits systematic investigation.

### 4.3 Antioxidant defense and osmotic adjustment

Antioxidant Defense and Osmotic Adjustment Reactive oxygen species (ROS) over-accumulation is a hallmark of drought stress, and the capacity to scavenge ROS is strongly correlated with drought tolerance (**Figure 1**). Several proteins have been shown to bolster antioxidant defense mechanisms in grapevine (Giordano et al., 2016; Yu et al., 2021; Cui et al., 2023; Lan et al., 2024). For instance, *VyRCHC114*, a RING-type E3 ubiquitin ligase isolated from Chinese wild grape (*Vitis pseudoreticulata*), exhibits E3 ubiquitin ligase activity *in vitro*. Its overexpression in Arabidopsis significantly improved drought tolerance, accompanied by enhanced activities of antioxidant enzymes and upregulation of stress-responsive genes (Yu et al., 2021). Similarly, *VyUSPA3*, a member of the universal stress protein A (USPA) family identified in *Vitis yeshanensis*, confers drought tolerance when overexpressed in transgenic grapevine. Transgenic lines displayed reduced stomatal aperture, improved root architecture, higher leaf relative water content, increased proline accumulation, elevated activities of antioxidant enzymes, and decreased levels of MDA, H_2_O_2_, and O_2_^-^ (Chen et al., 2021). Meanwhile, ROS also act as second messengers that regulate SnRK2 activity, amplifying the stress response (Mimata et al., 2026). Transcriptional upregulation of antioxidant genes by TFs enhances ROS scavenging, underpinning the reduced oxidative damage observed in drought-tolerant genotypes (Xu et al., 2025; Tan et al., 2025; Niu et al., 2023).

## 5 Conclusions and future perspectives

Substantial progress has been achieved in elucidating grapevine drought tolerance across multiple biological scales-from the molecular to the whole-plant level. At the molecular level, abscisic acid (ABA) signaling is well established as a central regulatory hub, with its core components functionally characterized. Hydraulic signals-not xylem embolism-mediate the initial closure of stomata (Tombesi et al., 2015; Albuquerque et al., 2020), whereas ABA sustains long-term stomatal regulation. A sophisticated transcriptional regulatory network has been delineated, wherein members of the NAC, WRKY, MYB, and bZIP transcription factor families collectively contribute to antioxidant defense activation and osmotic adjustment, yet exhibit distinct regulatory directions and target pathway specificities. Notably, WRKY transcription factors display the greatest functional diversity, encompassing both positive and negative regulators; in contrast, NAC, MYB, and bZIP family members are predominantly positive regulators. Their downstream target genes enhance drought tolerance through coordinated modulation of key physiological processes, including antioxidant defense, osmotic adjustment, and hormone metabolism.

At the whole-plant level, hydraulic vulnerability segmentation confers protection to perennial stems by preferentially sacrificing petioles and basal leaves (Hochberg et al., 2016, 2017). Root system adaptations exhibit remarkable inter-genotypic variation, including deep-root proliferation (Alsina et al., 2011), formation of cortical lacunae (Cuneo et al., 2021), suberin deposition in root tissues (Zhang et al., 2020), and xerophyte-like structural reinforcement of root architecture (Barrientos-Sanhueza et al., 2024). Furthermore, root traits and rootstock effects are increasingly acknowledged as critical determinants of grapevine drought adaptation. Drought-tolerant rootstocks display higher root hydraulic conductivity and a deeper root architecture, coupled with more rapid physiological recovery following rewatering (Alsina et al., 2011). Although grafting experiments robustly demonstrate that rootstocks exert profound influence on scion physiology (Cuneo et al., 2021), the identity and function of mobile signaling molecules mediating this cross-graft communication remain largely elusive-though emerging evidence implicates drought-responsive microRNAs (miRNAs) as key mobile regulators traversing the graft union (Pagliarani et al., 2017).

Despite these advances, several critical knowledge gaps persist. Field-scale validation is urgently required for both rootstock-mediated strategies (Nerva et al., 2022) and exogenous hormone applications (Wang et al., 2021; Zeng et al., 2024) to rigorously evaluate their efficacy across diverse edaphic, climatic, and management conditions. Moreover, the molecular mechanisms underpinning rootstock–scion communication-and the extent to which greenhouse-derived insights can be reliably extrapolated to field settings-remain insufficiently characterized.

Future research should prioritize three directions. First, integrating multi-omics datasets with high-resolution phenotyping and computational modeling will enable reconstruction of condition-specific gene regulatory networks. High-quality reference genomes for multiple Vitis species, complemented by transcriptomic, proteomic, and metabolomic resources (Haider et al., 2017; Savoi et al., 2017; Ma et al., 2023), provide a foundation for this effort. Machine learning applied to these datasets holds promise for uncovering emergent, system-level properties inaccessible through single-gene analyses. Second, advances in non-invasive root imaging will illuminate root architecture and function in drought resilience (Bernardo et al., 2025), facilitating selection of root traits optimized for diverse soil and climatic environments. Third, systematically introgressing validated drought-tolerance genes into elite backgrounds via marker-assisted selection or genome editing, coupled with strategic deployment of optimized rootstocks, represents the most sustainable pathway toward developing grapevine cultivars that concurrently maintain high productivity and premium fruit quality.
